# NetRank Recovers Known Cancer Hallmark Genes as Universal Biomarker Signature for Cancer Outcome Prediction

**DOI:** 10.3389/fbinf.2022.780229

**Published:** 2022-03-23

**Authors:** Ali Al-Fatlawi, Nazia Afrin, Cigdem Ozen, Negin Malekian, Michael Schroeder

**Affiliations:** Biotechnology Center (BIOTEC), Center for Molecular and Cellular Bioengineering, Technische Universität Dresden, Dresden, Germany

**Keywords:** cancer, network bioinformatics, hallmarks, biomarker, gene expression, microarray

## Abstract

Gene expression can serve as a powerful predictor for disease progression and other phenotypes. Consequently, microarrays, which capture gene expression genome-wide, have been used widely over the past two decades to derive biomarker signatures for tasks such as cancer grading, prognosticating the formation of metastases, survival, and others. Each of these signatures was selected and optimized for a very specific phenotype, tissue type, and experimental set-up. While all of these differences may naturally contribute to very heterogeneous and different biomarker signatures, all cancers share characteristics regardless of particular cell types or tissue as summarized in the hallmarks of cancer. These commonalities could give rise to biomarker signatures, which perform well across different phenotypes, cell and tissue types. Here, we explore this possibility by employing a network-based approach for pan-cancer biomarker discovery. We implement a random surfer model, which integrates interaction, expression, and phenotypic information to rank genes by their suitability for outcome prediction. To evaluate our approach, we assembled 105 high-quality microarray datasets sampled from around 13,000 patients and covering 13 cancer types. We applied our approach (NetRank) to each dataset and aggregated individual signatures into one compact signature of 50 genes. This signature stands out for two reasons. First, in contrast to other signatures of the 105 datasets, it is performant across nearly all cancer types and phenotypes. Second, It is interpretable, as the majority of genes are linked to the hallmarks of cancer in general and proliferation specifically. Many of the identified genes are cancer drivers with a known mutation burden linked to cancer. Overall, our work demonstrates the power of network-based approaches to compose robust, compact, and universal biomarker signatures for cancer outcome prediction.

## Introduction

Cancer is an uncontrollable growth of cells that can occur in nearly any organ of the human body. Biomarkers help to improve cancer diagnosis and disease progression. A number of biomarkers are in clinical use today, such as the Carbohydrate antigen 19-9 (CA19-9) for early detection of pancreatic cancer (Koprowski et al., 1981), MYC for monitoring the prognosis of lymphoma and leukemia, and *ALK,* for lung cancer (Targeted Cancer Therapies Fact Sheet—National Cancer Institute, 2021). Identifying highly accurate biomarkers is a complex problem. CA19-9, for example, is well established in pancreatic cancer but has only an accuracy of 70–80% (Al-Fatlawi et al., 2021), which means that it is not suitable for diagnosis on its own, but only to monitor relapse after surgery. One way to improve accuracy and robustness of the diagnoses is to employ biomarker signatures instead of only using single biomarkers. Key enabling technology for discovering biomarker signatures is high-throughput screening techniques such as microarray and deep sequencing. When microarrays were introduced in the late 90s, a first high-impact study identified a biomarker signature of 70 genes to estimate metastases after breast cancer surgery (van ’t Veer et al., 2002). Today, this signature is commercially available, and it is in wide use internationally as MammaPrint.

Defining such a signature is a complex undertaking as there are three requirements

1) Robustness: A signature should be robust to changes in the data, and it must not be over-optimized for a specific dataset. If the signature is applied independently to a dataset of a similar phenotype, it should perform similarly to the original dataset. If not, it could be overfitted and biased towards the original data.
2) Compactness: A signature should be compact. If a signature consists of thousands of genes, it becomes complicated to understand how individual components of the signature contribute to the prediction result.
3) Interpretability: A signature should be meaningful and interpretable. The identified genes should be connected to cancer, so that first steps can be taken to extend the correlation between biomarker and phenotype towards a causal model that explains how the biomarker links to the observed phenotype.

In general, discovering a biomarker signature for specific cancer and prediction is a daunting task due to combinatorial explosion. If a genome screen obtains data for 20,000 genes and a signature consists of 50 genes, then there are around 3.5 * 10^150^ possible signatures [C (20,000, 50) = 3.5 * 10^150^]. The vast majority of these signatures will not be suitable for any outcome prediction task. However, even if only a small percentage of signatures are suitable, it is still a large number. Consequently, many good signatures may exist. The breast cancer signature introduced by van’t Veer was complemented by a completely different signature for the same task with similar performance (Paik et al., 2004; Ein-Dor et al., 2006). This begs the question of how arbitrary the choice of a good signature could be.

Should not one expect that biomarker signatures for similar cancer types and outcome prediction tasks share some similarities? This should be especially true as different cancers share basic mechanisms such as survival, tumor growth, invasion, and others (Hanahan and Weinberg, 2011). These principles were summarized by Hanahan and Weinberg as hallmarks of cancer (Hanahan and Weinberg, 2000; Hanahan and Weinberg, 2011). They represent the biological properties acquired during the multistage development of cancer, including sustaining proliferative signaling, evading growth suppressors, resisting cell death, and seven other principles. Linking biomarkers to the hallmarks of cancer is one possibility for an interpretable signature. This paper defines a universal biomarker signature for cancer outcome prediction, which is robust, compact, and interpretable by pursuing a network-based approach.

There is a long-standing tradition to use interaction networks in biomarker discovery. Shi et al. developed a network-based signature for colorectal cancer recurrence by integrating several signatures and interaction networks (Shi et al., 2012). They highlighted the dysregulated biological processes in colorectal cancer recurrence. Dutkowski and coworkers combined gene expression profiles and physical protein interaction maps of embryonic tissue, metastatic breast cancer, and brain tumors to provide global network modules pointing out representative development and cancer programs (Dutkowski and Ideker, 2011). Winter et al. (Winter et al., 2012) developed a network-based outcome prediction approach—NetRank and successfully predicted patient survival using gene expression data. It ranks genes according to their network connectivity and statistical relevance using a modified formula for Google’s PageRank algorithm. NetRank was also applied to several cancer microarray datasets using transcription factor and protein-protein interaction networks (Roy et al., 2014). The study showed that integration of network information and gene expression data provides more accurate outcome predictions than classical methods on a par with signatures published by the authors of the studies. Barter et al. used gene expression microarray data from melanoma and ovarian cancer to predict patient clinical outcomes through gene expression. They compared three feature selection methods, including the most commonly used single gene (based on differential gene expression differences), gene-set (based on biological pathway or function), and network-based approaches (based on protein-protein interactions). The study also evaluated two network-based feature-selection algorithms: NetRank and GeneRank. As a result, they reported that NetRank was the most accurate for identifying more stable gene expression signatures (Barter et al., 2014).

**FIGURE 1:**
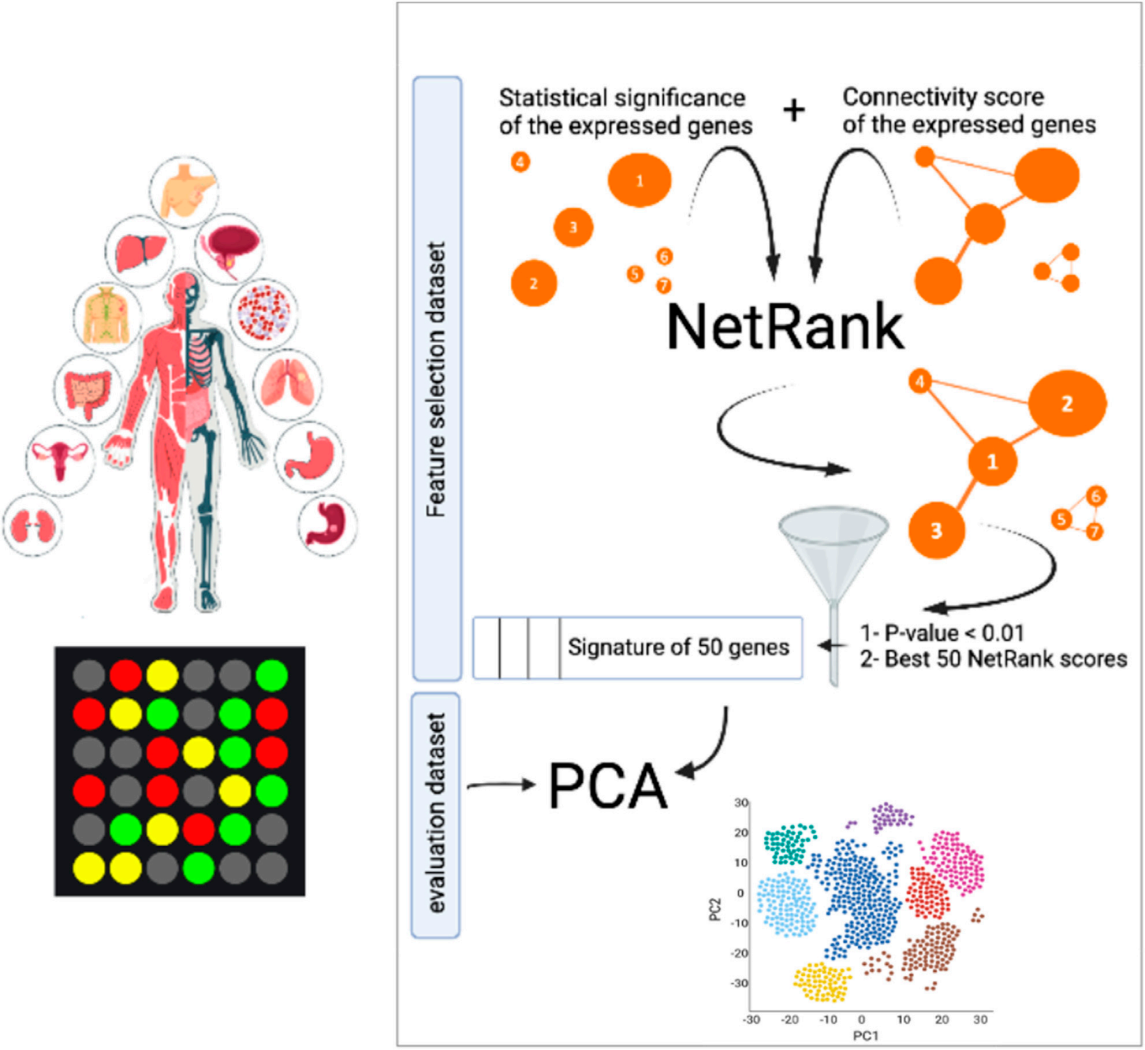
Overview biomarker discovery pipeline. NetRank identifies biomarker signatures by combining protein interactions from the String database with gene expression data. NetRank is applied to each dataset individually. Every dataset was split into a feature selection set (70%) and an evaluation set (30%). NetRank was applied to the first set. Principal component analysis was performed on the latter set using the selected features to evaluate the signatures in predicting the phenotype in an independent set.

We set out in this paper to collect 105 datasets covering 13 cancer types with different phenotypes. We proceed as depicted in the graphical abstract of Figure 1. In the first step, we show that biomarker signatures proposed by authors of the datasets do not overlap, and hence they follow the pattern that was already observed two decades ago when the two main breast cancer signatures turned out to be entirely different. Next, we develop our network-based approach by applying NetRank to the gene expression and phenotype data using the String database network (Szklarczyk et al., 2019), which covers over four million interactions between more than 20,000 proteins. We evaluate the performance of these signatures and compare the composition of these against each other and against the signatures originally proposed by the authors of the datasets. In the last step, we combine the NetRank biomarker signatures of each dataset into a global NetRank signature using majority voting. We evaluate the performance of this signature in terms of area under the curve for the cancer outcome prediction tasks and in terms of their relation to the hallmarks of cancer using an evaluation set. Overall, we show that the NetRank signature is robust, compact, and interpretable.

## Methods

### Datasets

**FIGURE 2:**
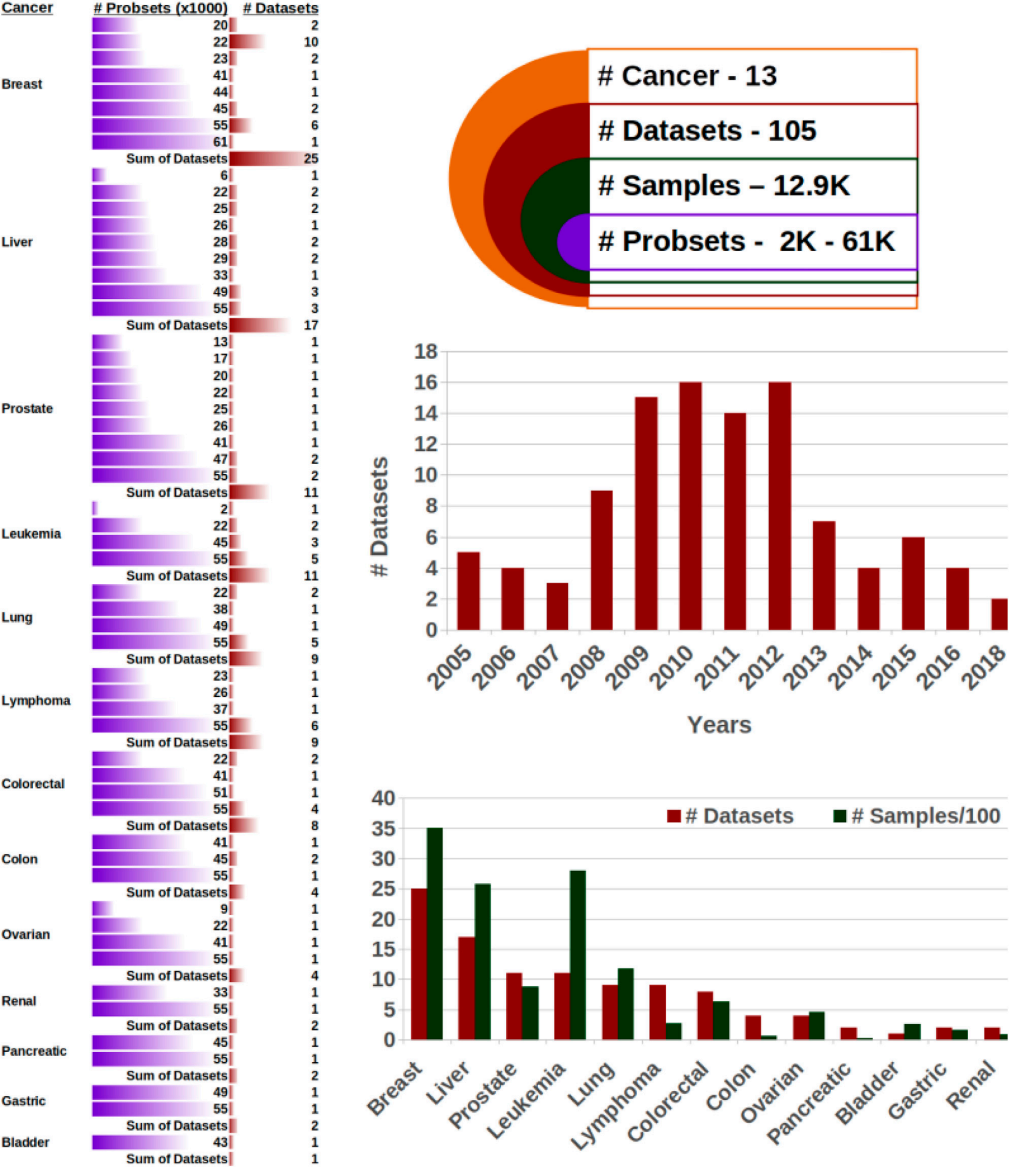
Microarray datasets. The 105 datasets are comprehensive, with around 13,000 samples from 13 cancer types, which each comprise a substantial number of probe sets.

Microarray datasets were obtained as follows. PubMed was queried in January 2021 for the keywords cancer and gene expression. Dates were limited to 2000 to 2020. To obtain only high-quality datasets, we filtered articles by impact factor greater than 15 and obtained ca. 3,700 papers. These were scanned manually for relevance to differential gene expression leaving 1,288 articles. For these, we found 225 datasets in the Gene Expression Omnibus database (Edgar et al., 2002). We filtered out 120 datasets because of their missing phenotype data (48), missing prob signals or few probes (35), missing gene symbols (11), the small size of fewer than six samples, or low quality indicated by many missing or NaN values (26). As a result, we kept 105 datasets. As demonstrated in Figure 2, the selected datasets comprise around 13,000 individuals for 13 cancer types with different phenotypes; Supplementary Table S1 and Supplementary Sheets S1, S2. Each dataset was normalized, evaluated, and studied individually, and then their outcomes were compared. Individuals of 11 out of the 105 datasets were mice, so we humanized their gene symbols using the R package biomaRt (Durinck et al., 2005).

### Microarray Data Processing

The robust multi-array averaging (RMA) method was used for background correction and normalization of the unnormalized datasets using the affy package in R 3.6 (Gautier et al., 2004). Affymetrix probes were mapped to gene symbols using the provided functional annotation of each dataset. We excluded the genes and samples with NaN values using the function “goodSamplesGenes” provided by the WGCNA R package 1.6.9 (Langfelder and Horvath, 2008), which kept only the records that have a minimum fraction of non-missing samples for a gene of 50%. We performed hierarchical cluster analysis and principal components analysis to evaluate the distance between individuals and remove the detected outliers using the R package stats 3.6.2 (R Core Team., 2020). Finally, the Pearson standard correlation coefficient and Fisher’s asymptotic *p*-value were determined using a robust correlation measure implementation (Langfelder and Horvath, 2008) in the R package WGCNA 1.6.9.

### Protein-Protein Interaction Network

To calculate the connectivity of each protein, we used the protein-protein interaction (PPI) STRING network (Szklarczyk et al., 2019). The analysis was carried out using the R package STRINGdb_1.26-0 with database version 10. The STRING database contains above four Mio interactions for more than 20,000 human proteins and above five Mio interactions for more than 22,000 mouse proteins. We have not applied any filtering for the connections. The nodes’ connectivity scores were normalized by dividing it by the maximum possible connectivity score in the network.

### NetRank

Our method is derived from the PageRank algorithm, which Google uses to rank web pages in their search engine. NetRank assumes a random surfer who navigates through a network of web pages by following links with probability *d* or starting new sessions on a random page with a probability of *1−d*. The random surfer visits a web page and randomly clicks on a link visiting the next page. Consequently, pages, which are central and well-connected, are visited more frequently by the random surfer than pages on the periphery of the network.

While PageRank takes only the node connectivity into account to designate ranking (Eq. 1), NetRank takes into account both connectivity and statistical association of the genes with a specific phenotype (Eq. 2).

$$r_{j}^{n}=\left(1-d\right)+d\overset{N}{\underset{i=1}{\sum }}\frac{m_{ij}\;r_{i}^{n-1}}{\deg ree_{i}},1\leq j\leq N$$ (1)

$$r_{j}^{n}=\left(1-d\right)s_{j}+d\overset{N}{\underset{i=1}{\sum }}\frac{m_{ij}\;r_{i}^{n-1}}{\deg ree_{i}},1\leq j\leq N\;$$ (2)

r: the node (gene) ranking score

n: iteration

j: index of the current node

d: damping factor (ranging between 0 and 1)

s: Pearson correlation coefficients

degree: the sum of the output connectivities for connected nodes

N: number of the total nodes

m: connectivity of connected nodes, $m_{ij}=1,\;$ if *i* and *j* are connected and 0 otherwise.

In Eq. 2, the damping factor d balances the impact of the network links (connectivity of the protein) with its statistical significance. In our implementation, we kept the value of d fixed (0.5) in all datasets to avoid any bias of having customized parameters in each analysis.

Here, the random surfer model is applied to an interaction network [such as the protein-protein interaction (PPI) network of STRINGdb (Szklarczyk et al., 2019)]. It combines the connectivity score with another score representing the correlation to the phenotype. Instead of counting page visits as in PageRank, NetRank initializes scores as the gene’s correlation to the phenotype. When the surfer visits a node, its correlation is distributed in equal parts to its neighbors. Then, its score is updated with one contribution from the correlation and the other from the neighbors’ scoring.

NetRank can be seen as method average across a network. Instead of considering a value in isolation, it is combined with its neighbors’ scores. In other words, two pieces of information are required to rank the coding genes: a network of the interactions between the genes and their statistical significance of association with the phenotype. For evaluating the significance of association between a phenotype and gene expression, we determined their correlation. Fisher’s asymptotic *p*-value was determined using an approximation to the true distribution. The advantage of Fisher’s asymptotic *p*-value is that it is valid in small and large sample sizes (Agresti, 1992). A *p*-value of 0.01 or below was considered significant in the analysis. Supplementary Figure S1 shows the pseudo-code for this procedure.

### Data Splitting and Feature Selection

In our analysis, the NetRank algorithm was applied on only 70% of each dataset (feature selection set), and we kept 30% unseen for evaluating the approach (Figure 1). To keep the signatures compact and avoid bias toward specific datasets, we specified a threshold of 50 genes (maximum) for all datasets and selected those that showed the highest-ranking and met the *p*-value requirement below 0.01.

### Outcome Prediction Using PCA

Principal component analysis was performed using Python 3.7.6 with core functions provided by scikit-learn (sklearn) 0.20.3 (Pedregosa et al., 2011). It was applied only on the datasets with an adequate number of samples in each class in the test set (i.e., at least four samples per class). In our analysis, out of 105 datasets, 60 had enough samples in the test set for clustering (i.e., six samples). The area under the ROC curve (AUC) was calculated using scikit-learn for the best component in the PCA analysis.

### Cancer Hallmarks Genes

The selected genes in our signature were manually searched on the Cancer Hallmarks Genes (Zhang et al., 2020). Cancer Hallmarks Genes dataset has a collection of 2,940 genes that are categorized into ten hallmarks. We searched for our genes in each hallmark, and provided degree and betweenness centrality information of the gene in different hallmark networks.

### Biological Interpretation

We searched for possible existing drugs for our protein list in ChEMBL (Gaulton et al., 2017) using the open-target project (Koscielny et al., 2017) and provided the results. Moreover, we check these genes in the Cancer Genome Atlas Data Portal (GDC, 2021).

## Results

This study explores the possibility of a universal cancer signature arising from processes common to most cancers. Inspired by the hallmarks of cancer, it investigates whether mechanisms such as tumor growth or cancer survival and progression, which are present in all cancers, can give rise to biomarker signatures, which perform well in cancer outcome prediction tasks.

To this end, we devised a universal signature, which shows good performance across many types of cancer in the cancer outcome prediction tasks. The defined signature is compact interpretable in that its genes have confirmed links to cancer, the hallmarks of cancer in particular. The base for this goal is a large dataset of gene expression data for cancer outcome prediction tasks.

**105 datasets cover 13,000 samples, 13 cancer types, and over ten phenotypes.** When collecting datasets for our study, we had two goals: The collection had to be comprehensive, covering many types of cancer and various outcome prediction tasks, and the data had to be high quality. We addressed both aims by screening scientific literature and focusing on high-impact publications. After rigorous filtering as described in the methods section, we obtained 105 microarray datasets. These datasets ranged very substantially in size from some specialized, small-scale studies with as little as six samples (such as GSE73396 in liver cancer and GSE43444, GSE17538 in colon cancer) to a large-scale multi-center study to evaluate the use of microarrays in leukemia diagnosis with 2,096 samples (GSE13204). The average size per dataset is 73 samples. In total, there were 12,900 samples.

Overall, the samples were very diverse in terms of cancer types and phenotypes. The largest number of studies dealt with breast cancer (25), followed by liver (17) and prostate, leukemia, lung, and lymphoma with around ten each. Overall, 13 different types of cancer are present (Figure 2). The overwhelming majority of datasets consisted of human samples, and however, 206 of the 12,900 samples were from mice. The phenotypes investigated in the 105 studies also captured a broad range, including grading (18), distinctive cancer-specific phenotypes such as epithelial cell adhesion molecules in the liver and lymph node status in breast cancer (16), cancer vs. non-cancer (12), metastasis status or localization (12), subtypes (9), survival status or time (7), mutation of genes or receptor (6), treatment effect (4), tumor localization (4), remission or relapse (3), progression (1), and 13 others (Supplementary Sheets S2). This comprehensive mixture ensures that easier tasks such as distinguishing healthy from cancerous tissue as well as more complex tasks such as survival are present.

Nearly all studies used standard microarrays, and every of the 13 cancer types has at least two datasets with over 40,000 probes. Only three out of the 105 datasets have less than 10,000 probes. The dataset with the smallest number of probes (1,756) is also the largest dataset with 2,096 samples.

The datasets span a period of 13 years from 2005 to 2018, with peaks between 2009 and 2012, which is in line with the introduction of microarrays in the late 90s to early 00s and the recent advent of low-cost deep sequencing as a new technology superseding microarray.

We collected gene expression signatures that were proposed by the authors of the datasets. Taken together, the 105 author signatures comprise 4,343 genes. The signatures vary immensely in size with the smallest consisting of only one gene and the largest of 3,232 genes. The average number of genes put forward by the authors of the datasets is therefore 41 (4,343/105). This is a similar order of magnitude as the highly successful 70 gene signature underlying the Mammaprint breast cancer signature (van ’t Veer et al., 2002). Therefore, we fixed the size of signatures to be proposed by our methods to 50.

**FIGURE 3:**
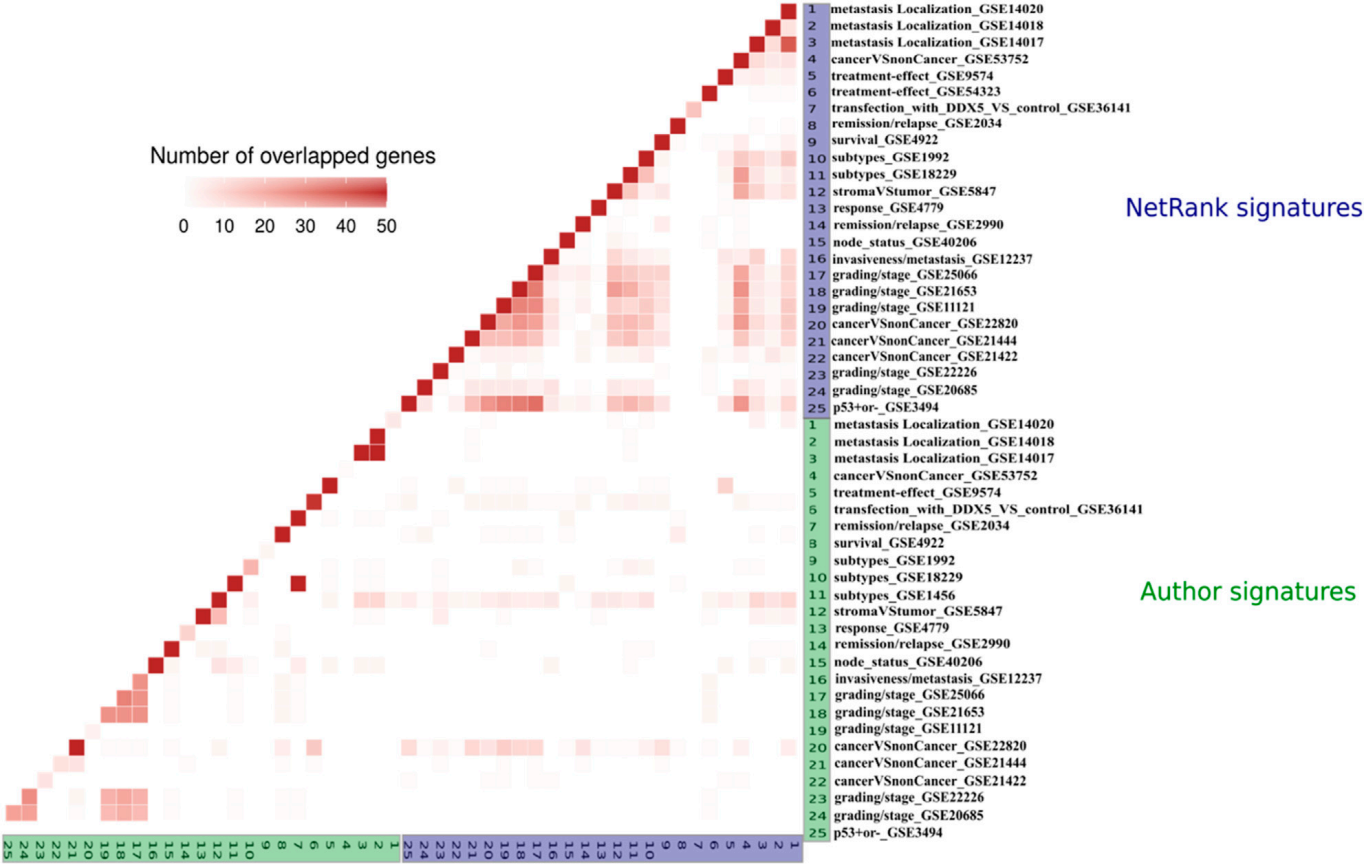
Overlap of the author (green) and the NetRank (blue) signatures for 25 breast cancer datasets. Author signatures do generally not overlap (green vs. green triangle in the bottom left). Author signatures hardly overlap with NetRank signatures (blue vs. green rectangle in the bottom right). NetRank signatures strongly overlap (blue vs. blue triangle in top right).

**Author signatures are dissimilar.** The starting point of our analysis is how similar or dissimilar biomarker signatures are across datasets and tasks. Given that we have 25 breast cancer datasets, one could expect that the signatures for these datasets overlap. The degree of similarity between the investigated phenotypes relates to the degree of overlap. As shown in Figure 3 and Supplementary Figure S2, the datasets hardly overlap. The only significant overlap exists between datasets from the same study (GSE25066, GSE21653, GSE11121, GSE20685, GSE21653, GSE3494) (Ko et al., 2013), which was a meta-analysis on the role of ion channels as predictors. We expanded these comparisons to all datasets (Supplementary Figure S3) and found the same: Author signatures hardly overlap, and this holds in particular for each of the 13 cancer types.

This finding was in agreement with the observation of Ein-Dor et al., who observed the lack of similarity between signatures of different studies for the same prediction task (Ein-Dor et al., 2006). While pathways in different tissues are formed from specific genes and proteins and while author signatures were introduced in this specific context, we aim to highlight the commonalities of data sets. Besides the significant contribution of the aforementioned signatures in underlying the genetic information in each cancer and phenotype type individually, there is a necessity for studying the shared genomic process and biological phenomena in cancer generally, regardless of particular cell types or tissue. This concept of generalization in principles of cancer biology is highly inspired by the notion of cancer hallmarks which helps in understanding the common mechanisms of tumor growth and cancer survival and progression.

**Standard correlation signatures are dissimilar.** One trivial reason why there is only so little overlap between the signatures proposed by the original authors of the datasets is that there may have been differences in pre-processing and normalizing the data and in the selection procedure for biomarkers. Therefore, we processed all datasets in the same manner (Section 2) and devised a simple method to generate a biomarker signature per dataset. We correlated each gene to the desired phenotype and combined the top 50 genes with the best correlation into a signature. Supplementary Figure S4 shows the pairwise overlap between these signatures. Moreover, again, there is hardly any overlap.

**NetRank signatures are similar.** To focus on the common cancer characteristics, we employed a network-based approach which added a new aspect to the biomarker selection process. It combines two forms of information in ranking biomarkers: first, the gene’s correlation to the target phenotype as introduced above; second, the interactions between these genes (Section 2).

After running NetRank on a dataset, we define the top 50 genes with the highest NetRank score and *p*-value lower than 0.01 as NetRank signature for this dataset. Strong overlap was found between signatures of the same cancer type (see the overlap of breast cancer signatures in Figure 3 and Supplementary Figure S5). Considerable overlap was noted even between different cancer types (Supplementary Figure S6). We specify the 50 most overlapped biomarkers within each cancer as a signature for that cancer. Supplementary Sheets S3 present the signatures of 13 cancer types used in further analysis to propose a universal cancer signature.

**NetRank is an outstanding feature selection technique.** For each dataset, we created a feature selection set (70%), which NetRank uses, while 30% were kept unseen to serve as an evaluation set (Figure 1). In the evaluation process, to avoid over-optimization of the outcome prediction with the signatures, we used a linear dimension reduction technique (PCA) instead of more complex non-linear methods such as machine learning with neural networks. Using the independent evaluation set, we evaluated the features by calculating the area under the ROC curve (AUC) of each dataset’s best principal component in the PCA analysis. The closer the AUC to 1, the better the predictive model.

Figure 5 and Supplementary Sheets S2 show that 74% of the datasets were classified with AUC better than 0.80. Thus, NetRank serves as an outstanding feature selection method in bringing biologically meaningful features without causing a considerable drop in performance.

We compared the performance of the NetRank’s features with those chosen by the standard correction method. Statistically, the standard correlation features performed slightly better (78% of the datasets having AUC better than 0.80 compared to 74% for the NetRank features). Importantly, NetRank features were highly overlapped and biologically relevant.

**FIGURE 4:**
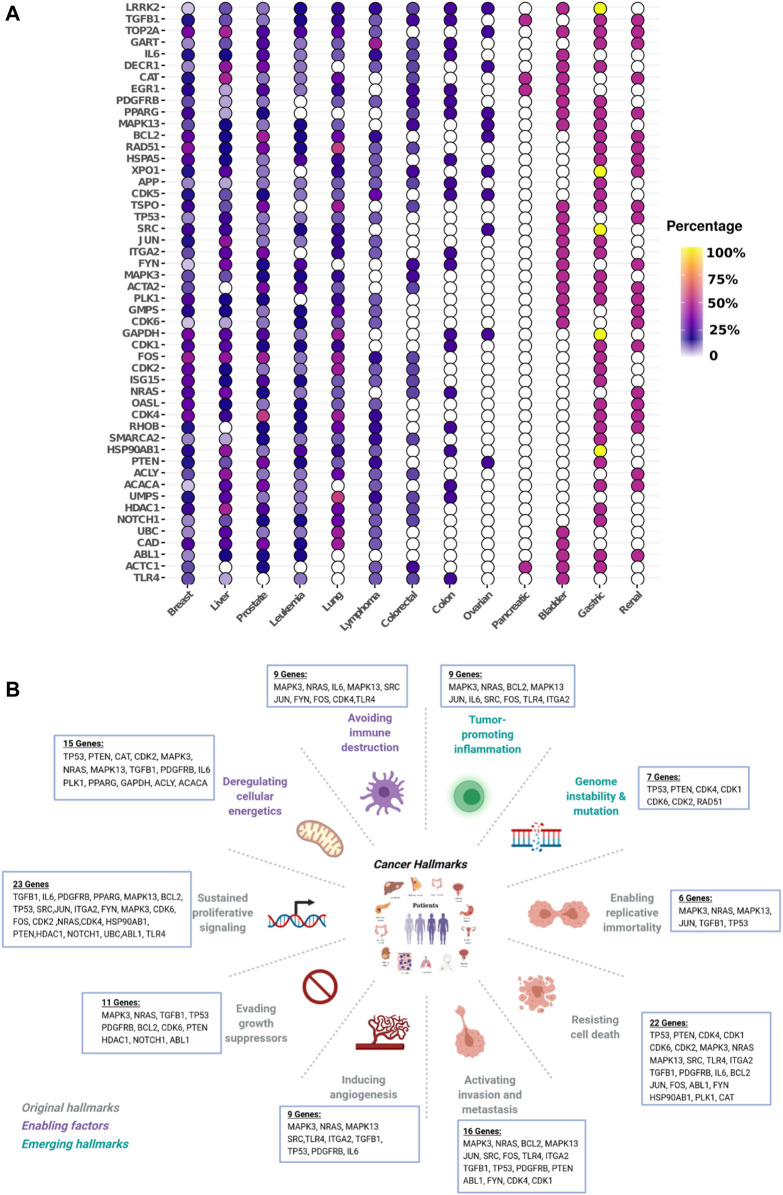
Universal NetRank signature in different cancer and hallmarks. Top 50 most frequent genes in the 105 NetRank signatures. **(A)**: Gene vs. cancer type. The color indicates how frequently the gene was part of a NetRank signature. All genes were selected as biomarkers in several cancer types. Pancreatic cancer stands out with hardly any genes present. **(B)**: Break down the 50 genes of the universal NetRank signature by ten hallmarks of cancer. All hallmarks are captured by the signature.

**Compact and robust universal biomarkers signature.** Given the strong overlap between the 13 NetRank signatures illustrated in Supplementary Figure S6, we asked whether it is possible to combine individual NetRank signatures for each dataset into one universal NetRank signature for all datasets. We took a consensus approach. We counted how often each of our genes was selected in any of the 13 NetRank signatures. We defined the universal biomarker signature as the top 50 genes, which appear most frequently in any NetRank signature. These 50 biomarkers are illustrated in Figure 4. Except for pancreatic and ovarian cancer, they were associated with all types of cancer.

With 50 genes, the universal signature is compact, which leaves the question of whether it is robust. The biomarkers in the universal signature are special. Due to the network-based approach, they comprise central and well-connected genes in the protein interaction network. They emerged from a consensus method and should therefore be widely applicable. To assess robustness, we had to evaluate the predictive power of the universal signature and define a baseline as a comparison. As a baseline, we selected the standard correlation signature and the NetRank signature. Since both are optimized for a dataset, we expect the universal signature to perform less well than these two signatures.

We applied dimension reduction to the evaluation sets (30%) using the 50 features of the universal biomarker signature. Then we evaluated again by calculating the area under the ROC curve (AUC). Given that biomarkers such as CA19-9, which is widely used in pancreas cancer diagnosis, achieve 70–80%, we consider an AUC of 0.80 success.

**FIGURE 5:**
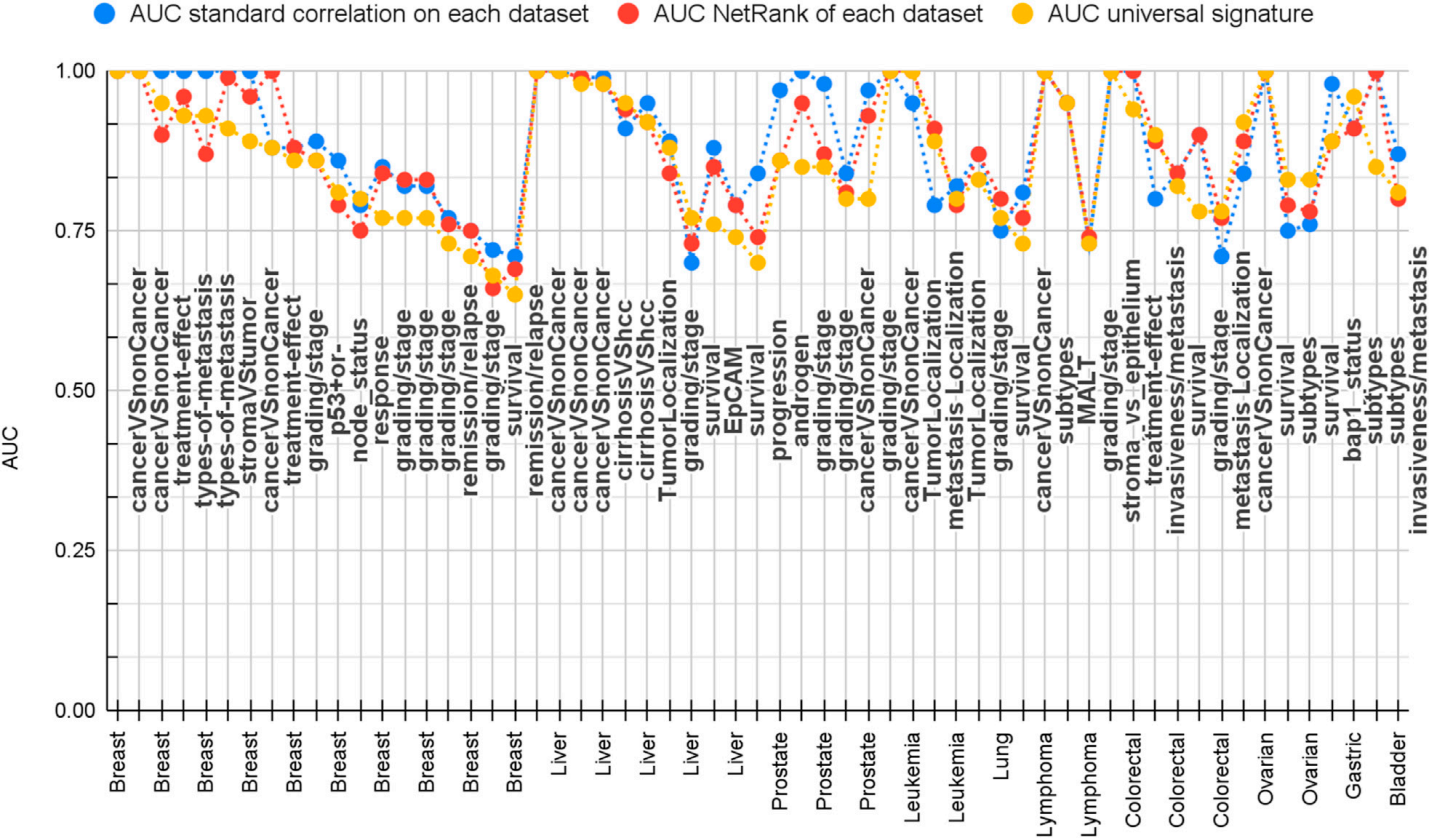
Comparison of prediction performance of correlation (blue), NetRank (red), and universal (yellow) NetRank signature. 105 datasets vs. performance measured as AUC. All signatures achieve across all datasets a good AUC. The universal signature has a comparative performance to the correlation and NetRank signature, which are optimized per dataset. All results can be found in Supplementary Sheets S2.

Overall, we found that the correlation signature has this successful performance for 78% of datasets, the NetRank signatures for 74%, and the universal signature for 66% (Figure 5 and Supplementary Sheets S2). A closer inspection reveals that predicting survival time, disease grades, and progression was more difficult than distinguishing cancer from control. Most of the cases that have AUC below 0.80 were for these phenotypes (Figure 5). In contrast, cancer versus non-cancer can be very well separated.

Overall, all three approaches produce satisfactory statistical results for the majority of datasets. The key difference resides in the number of different genes that are necessary. Across all 105 datasets, the correlation signatures consist of 3,812 different genes, which is close to the 4,343 genes proposed in total by the authors of the datasets. For each test, we used 50 of them that are optimized for that dataset. In contrast, the union of all 105 NetRank signatures has already a reduced size of 1,770 genes, and by definition, the universal signature comprises only 50 genes. Therefore, the universal signature is a compact condensation of the key genes’ performant across all data.

**The universal biomarker signature relates to commercially available signatures.** First, the protein-protein interactions between these biomarkers indicate their high connectivity and possible functional interaction in biological processes (Supplementary Figure S7). We compared our universal signature with currently used tumor signatures as well. We found three genes (PLK1, TOP2A, RAD51) are in common with Prolaris prostate cancer signature (Crawford et al., 2014), two genes (BCL2 and GAPDH) with the breast cancer signature Oncotype Dx (Paik et al., 2004), other two genes (TP53, PTEN) with ColoNext (Colon Cancer Genetic Testing, 2021), one gene (GMPS) in common with the breast cancer signature Mammaprint (van ’t Veer et al., 2002), and finally one gene (BCL2) with the Prosigna breast cancer test PAM50 (Parker et al., 2009).

**The universal biomarker signature recovers known cancer hallmark genes**. It is interpretable in the sense that it connects well to the hallmarks of cancer, although this information was not used to generate the universal signature. To assess this connection, we used the Cancer Hallmarks Genes (CHG) database designed by Zhang et al. (Zhang et al., 2020), which comprises 2,940 genes and their association to one or more hallmarks. We searched the 50 genes in the universal signature and found that 31 are listed in the hallmarks database (Table 1). From the perspective of hallmarks, at least six genes in the universal signature represented each of the ten hallmarks. The most strongly represented hallmarks were “sustaining proliferative signaling” (23 genes), “resisting cell death” (22 genes), and “activating invasion and metastasis.” (16 genes). At the level of individual biomarkers, we found the five biomarkers TGFβ1, MAPK13, TP53, MAPK3, and NRAS in at least seven hallmarks and at least eight cancer types. They play well-defined roles in particular cancers such as breast, liver, lung, melanoma (Zarzynska, 2014; Cicenas et al., 2017; Silwal-Pandit et al., 2017; Afrăsânie et al., 2019; Lee et al., 2020; Stolfi et al., 2020). BCL2, as another example. Its gene rearrangements are used for diagnosing and planning Lymphomas and Leukemias (Targeted Cancer Therapies Fact Sheet—National Cancer Institute, 2021). In our analysis, BCL2 was found in nine cancers and involved in five hallmarks. Furthermore, considering hallmark types and numbers, MAPK3 and NRAS showed the same profile, and they were involved in 9 out of 10 cancer hallmarks. We have provided degree and betweenness centrality information of the genes of universal signature in different hallmark networks in Supplementary Table S2.

**TABLE 1:** Universal NetRank signature genes and the hallmarks of cancer. Cancer Hallmarks: 1: Sustaining proliferative signaling; 2: Evading growth suppressors; 3: Evading immune destruction; 4: Enabling replicative immortality; 5: Tumor-promoting inflammation; 6: Activating column and metastasis; 7: Inducing angiogenesis; 8: Genome instability and mutation; 9: Resisting cell death; 10: Reprogramming energy metabolism. “# Cancer” parameter indicates how many cancers a particular gene was found in the analysis. “√” means that the gene (row) is involved in one pathway of a specific hallmark of cancer (column). SUM shows how many genes are involved in a particular cancer hallmark.

Gene symbol	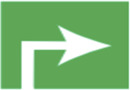 1	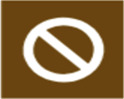 2	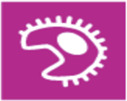 3	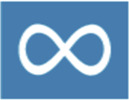 4	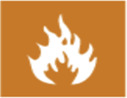 5	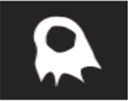 6	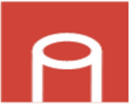 7	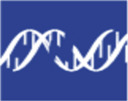 8	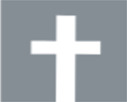 9	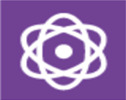 10	# Cancer
LRRK2	—	—	—	—	—	—	—	—	—	—	11
TGFB1	√	√	—	√	—	√	√	—	√	√	11
TOP2A	—	—	—	—	—	—	—	—	—	—	10
GART	—	—	—	—	—	—	—	—	—	—	10
IL6	√	—	√	—	√	—	√	—	√	√	9
DECR1	—	—	—	—	—	—	—	—	—	—	9
CAT	—	—	—	—	—	—	—	—	√	√	9
EGR1	—	—	—	—	—	—	—	—	—	—	9
PDGFRB	√	√	—	—	—	√	√	—	√	√	9
PPARG	√	—	—	—	—	—	—	—	—	√	9
MAPK13	√	—	√	√	√	√	√	—	√	√	9
BCL2	√	√	—	—	√	√	—	—	√	—	9
RAD51	—	—	—	—	—	—	—	√	—	—	9
HSPA5	—	—	—	—	—	—	—	—	—	—	9
XPO1	—	—	—	—	—	—	—	—	—	—	9
APP	—	—	—	—	—	—	—	—	—	—	9
CDK5	—	—	—	—	—	—	—	—	—	—	9
TSPO	—	—	—	—	—	—	—	—	—	—	8
TP53	√	√	—	√	—	√	√	√	√	√	8
SRC	√	—	√	—	√	√	√	—	√	—	8
JUN	√	—	√	√	√	√	—	—	√	—	8
ITGA2	√	—	—	—	√	√	√	—	√	—	8
FYN	√	—	√	—	—	√	—	—	√	—	8
MAPK3	√	√	√	√	√	√	√	—	√	√	8
ACTA2	—	—	—	—	—	—	—	—	—	—	8
PLK1	—	—	—	—	—	—	—	—	√	√	8
GMPS	—	—	—	—	—	—	—	—	—	—	8
CDK6	√	√	—	—	—	—	—	√	√	—	8
GAPDH	—	—	—	—	—	—	—	—	—	√	8
CDK1	—	—	—	—	—	√	—	√	√	—	8
FOS	√	—	√	—	√	√	—	—	√	—	8
CDK2	√	—	—	—	—	—	—	√	√	√	8
ISG15	—	—	—	—	—	—	—	—	—	—	8
NRAS	√	√	√	√	√	√	√	—	√	√	8
OASL	—	—	—	—	—	—	—	—	—	—	8
CDK4	√	—	√	—	—	√	—	√	√	—	8
RHOB	—	—	—	—	—	—	—	—	—	—	8
SMARCA2	—	—	—	—	—	—	—	—	—	—	8
HSP90AB1	√	—	—	—	—	—	—	—	√	—	8
PTEN	√	√	—	—	—	√	—	√	√	√	8
ACLY	—	—	—	—	—	—	—	—	—	√	8
ACACA	—	—	—	—	—	—	—	—	—	√	8
UMPS	—	—	—	—	—	—	—	—	—	—	8
HDAC1	√	√	—	—	—	—	—	—	—	—	8
NOTCH1	√	√	—	—	—	—	—	—	—	—	8
UBC	√	—	—	—	—	—	—	—	—	—	7
CAD	—	—	—	—	—	—	—	—	—	—	7
ABL1	√	√	—	—	—	√	—	—	√	—	7
ACTC1	—	—	—	—	—	—	—	—	—	—	7
TLR4	√	—	√	—	√	√	√	—	√	—	7
SUM	23	11	9	6	9	16	9	7	22	15	—

While 31 genes in the universal signature are linked to the hallmarks, 19 are not. We investigated the 19 further and found that four of these 19 genes are cancer drivers. It has been reported that LRRK2 (leucine-rich repeat kinase 2) plays an adaptive role between cancer and Parkinson’s disease and is defined as a new target molecule for cancer therapy due to its increased kinase activity (Warø and Aasly, 2018; Kim and Jeong, 2020). GMPS guanine monophosphate synthetase is in the Mammaprint gene list that composes known biomarkers for breast cancer defined by Tian et al. (Tian et al., 2010). Other examples were TOP2A and GART. TOP2A, as a DNA topology changer in various DNA associated processes (i.e., replication, chromosome segregation, recombination), is a well-known anti-cancer drug target. Almost 50% of chemotherapies include at least one of the TOP2A inhibitors such as etoposide or doxorubicin (Nitiss, 2009). GART gene is a trifunctional purine biosynthetic protein adenosine-3, a part of nucleotide metabolism, specifically purine metabolism. Cong et al. associated GART with poor prognosis in hepatocellular carcinoma and reported it as a liver cancer cell proliferation promoter (Cong et al., 2014).

The immediate and exciting result of the paper is that the use of network information helps select biomarkers, which represent the hallmarks of cancer, although this information was not explicitly used in the generation of the biomarkers. Overall, we have demonstrated that NetRank, which combines interaction, expression, and phenotype data, can generate robust, compact, and interpretable biomarkers signatures for cancer outcome prediction.

**The universal biomarker signature picks cancer drivers and drug targets.** Evaluating the genes in the universal signature using the Cancer Genome Atlas (TCGA) reveals that most have degrees of somatic mutations in different cancers. We found simple somatic mutation frequencies between 0.3% (TSPO) to 49.86% (TP53). In addition, we found 14 genes (TP53, PTEN, NOTCH1, NRAS, PDGFRB, ABL1, XPO1, HSP90AB1, PPARG, GMPS, JUN, CDK6, BCL2, CDK4) that are also cancer driver genes. Driver genes are defined as those genes that contain mutations that have been causally implicated in cancer and explain how dysfunction of these genes drives cancer) in the Cancer Gene Consensus database (Cancer Gene Census). These results can be viewed in Supplementary Sheets S2. Finally, some of our genes are already defined as drug targets for some types of cancer. We found that most clinical trials, completed or incomplete, targeted ABL1, BCL2, CDK1, CDK2, CDK5, FYN, PDGFRB, PLK1, TOP2 for various types of leukemia.

## Discussion and Conclusion

Biomarkers play a vital role in cancer diagnosis and treatment. Composing suitable biomarker signatures is a complex problem as it requires selecting a limited number of markers from a genome-wide screen. Subsequently, many biomarker signatures reported in the literature were context-specific and did not overlap. This is not surprising as the pathways in different tissues are formed from specific genes and proteins, and the author signatures were introduced accordingly. In this work, we aimed to study shared characteristics of different cancers, taking into account the shared core functions of cancer in different organisms, which were defined as hallmarks of cancer. The latter summarizes and groups these characteristics in ten principles, namely: sustaining proliferative signaling, evading growth suppressors, evading immune destruction, enabling replicative immortality, tumor-promoting inflammation, activating column and metastasis, inducing angiogenesis, genome instability and mutation, resisting cell death, reprogramming energy metabolism (Hanahan and Weinberg, 2011).

In this study, we addressed this imbalance and employed a network-based method, NetRank, to identify robust biomarkers, which perform across many cancer types and phenotypes. We adapted a random surfer model, which incorporates gene expression, large-scale interaction data, and phenotypic data from the 105 datasets into a feature selection model applied to the 105 datasets. The resulting biomarkers were aggregated and focused on the most frequently selected ones. The result is a universal biomarker signature of 50 genes, which is very compact in comparison to the total of 4,343 distinct genes proposed in signatures of the original data. Using PCA, the universal NetRank signature showed very strong prediction performance across nearly all cancer types except pancreas cancer and across all phenotypes. Thus, this signature is compact, robust, and performant, and it is linked to the hallmarks of cancer genes, although this information was not incorporated in the model. Over half of the genes in the NetRank signature are hallmark genes. Furthermore, a large number are cancer driver genes with a known mutation burden, and others are cancer drug targets. Thus, the use of networks in phenotype prediction leads to reliable, transferable, and interpretable biomarker signatures.

Pancreatic cancer and, to some extent, ovarian cancer are exceptions as they have only a few shared biomarkers with the other cancers. This is probably due to the complexity of the genetic component of pancreatic cancer, which makes it not easily explainable. It is widely accepted that other low penetrance genes play a role in pancreatic cancer (Milne et al., 2009; Klein and Westenberger, 2012; Al-Fatlawi et al., 2021). When we looked deeper into biomarker studies, including both pancreatic and ovarian cancer, we realized that both pancreatic and ovarian cancer are similar in terms of tissue structure, and both are located in the endocrine system. It has been reported that the biomarkers CA19-9 and CA125 are used to detect both cancer types and that many studies have reported complexity in their genetic components (Nolen and Lokshin, 2014). Unfortunately, a satisfactory explanation for what makes these two organs so special is still obscure (Ueland, 2017; Brezgyte et al., 2021). However, there is an interesting study on this subject: In 2019, Yeung and colleagues reported that both ovarian and pancreatic cancer are surrounded by cancer-associated fibroblasts (CAF), and CAF increases angiogenesis and metastasis in these cancers by releasing the microfibril-associated protein 5 (MFAP5) (Yeung et al., 2019). However, as can be seen, this protein is not directly related to these two cancers, at least at the level of the cellular transcriptome. This indicates that both ovarian and pancreatic cancer are affected by microenvironmental factors rather than intracellular factors. In this case, biomarker studies related to these two cancer types need to be examined in terms of microenvironmental factors also.

Regarding the data, our study includes the majority of human data and a few mouse datasets. While including mouse datasets does not significantly influence the study, it adds valuable information and an indication of the study’s replicability. Supplementary Sheets S2 shows that removing mouse datasets has no notable influence on the results, as human datasets mainly indicated the genes included in our universal signature. For example, our best five genes: LRRK2, TGFB1, TOP2A, GART, and IL6, were among the best 50 genes and were significant in 16, 18, 31, 17, and 17 human datasets, respectively, in comparison with only 4, 1, 3, 3, and 1 mouse datasets.

This study builds on biomarker signatures discovered over the last two decades from microarray data. This time period was necessary to turn these signatures into commercial products used in clinical practice. In the meantime, microarrays are superseded by deep sequencing techniques. It is interesting to explore our approach on RNA-Seq data. However, to date, microarray data is still much more abundant than RNA-Seq data. As an estimate for the ratio of microarray to RNA-Seq data, we queried PubMed for “microarray cancer outcome prediction” and for “deep sequencing cancer outcome prediction”. The former returned 19,000 papers, the latter 3,000. The former spread out over the two decades with 1,500 papers per year and a recent decrease due to the advent of RNA-Seq. The latter rises steeply peaking at 750 papers. In a few years time, there will be sufficient RNA-Seq data to perform a similar analysis on this type of data.

In summary, we have demonstrated that it is possible to compose biomarker signatures that build on common principles of cancer and subsequently perform well on many cancer types and prediction tasks. This universal signature may serve as a starting point and as one building block to develop highly optimised and precise signatures for specific cancer types and outcome prediction tasks.

## Data Availability

Publicly available datasets were analyzed in this study. This data can be found here: https://www.ncbi.nlm.nih.gov/geo/.
