# Supplementary material for: NetRank Recovers Known Cancer Hallmark Genes as Universal Biomarker Signature for Cancer Outcome Prediction
Source: Front Bioinform. 2022 Mar 23;2:780229. doi: 10.3389/fbinf.2022.780229 (PMC9580863; doi:10.3389/fbinf.2022.780229)
Supplement: Supplementary file 1 [file DataSheet4.docx]

**Supplementary I**

| **Inputs: features (probe signals), labels (phenotypes)**  **Outputs: feature rank (score) and p-value of association**  **Normalization & outlier detection:**   \|  \| 1- If data is not normalized:  features←RMA (features) # normalization  2- Filter samples and genes with high missingness or NaN values (using the function 'goodSamplesGenes')  3- Hierarchical cluster analysis on the samples using average tree  4- Filter outliers. \| \| --- \| --- \|   **NetRank:**   \|  \| 1- Determine the standard correlation coefficient (Pearson correlation).  2- Determine the fisher p-value of standard correlation  3- NetRank:  3.1 STRINGdb   - fetch string_db data for the provided proteins - Get interactions of each protein. - Divide each interaction connectivity by the max connectivity to normalize the values.   3.2 NetRank score  minError ← 0.01 # when the difference in the root mean square errors between iterations is below 0.01, then stop iterating  while rmsd > minError do  $r_{j}^{n}=(1-d)s_{j}+d\sum_{i=1}^{N} \frac{m_{ij}r_{i}^{n-1}}{{degree}_{i}}, 1\leq j\leq N$  $rmsd= r^{n}$-$r^{n-1}$  r: the node (gene product) ranking score  n: iteration  N: number of total nodes  j: id of the current node  d: damping factor (ranging between 0 & 1)  s: Pearson correlation with phenotype  m: connectivity of connected nodes (gene products)  degree: the sum of outputs for connected nodes (gene products)  end while  4- Feature selection:  4.1 Filter any feature with a p-value higher than 0.01  4.2 Keep 50 features with the highest score (NetRank score). \| \| --- \| --- \|   **Figure S1. Our workflow: NetRank algorithm.** |
| --- | --- | --- | --- | --- |

| **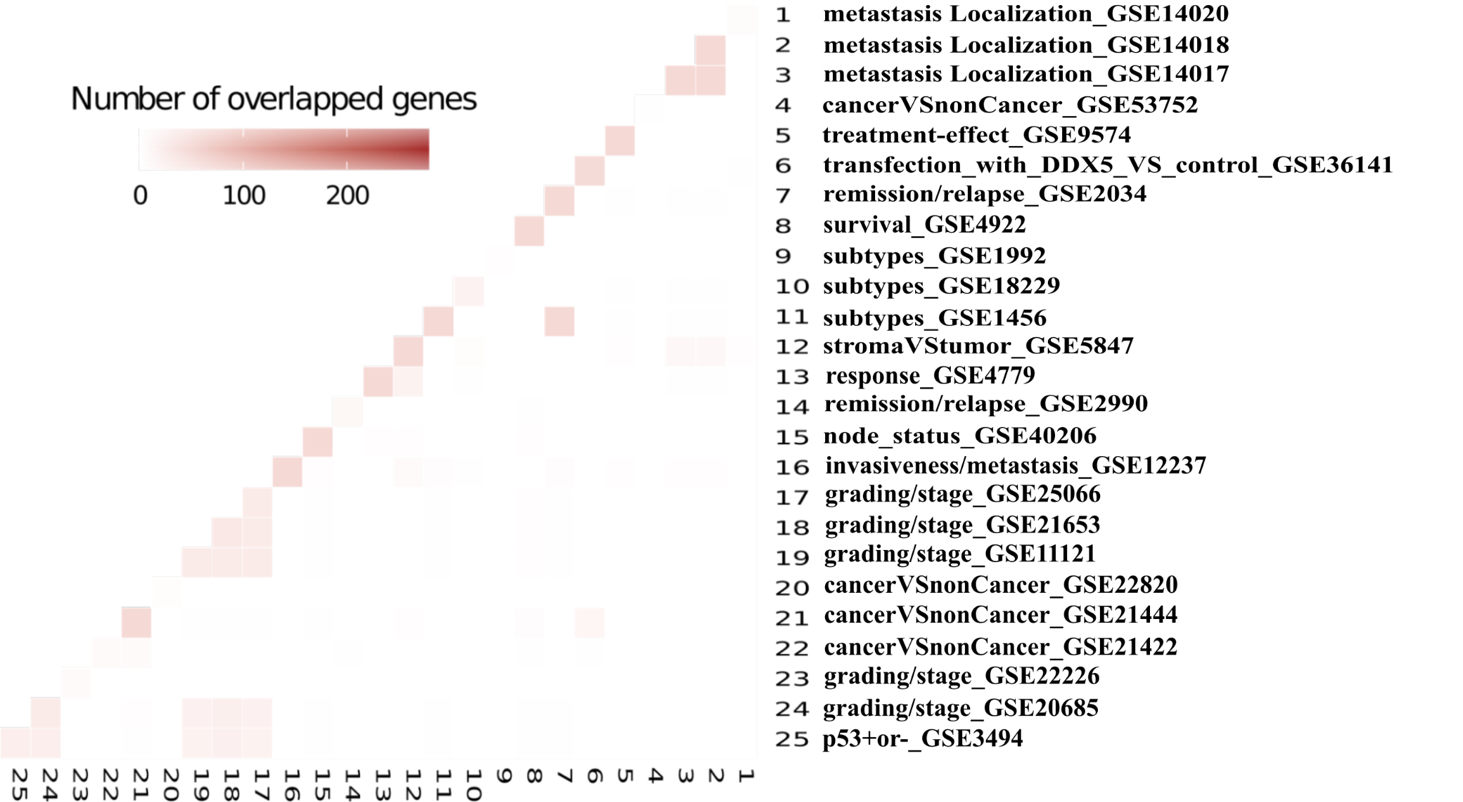** |
| --- |
| **Figure S2. Heatmap of collected author signatures for breast cancer datasets.** Heatmap showing overlapping gene signatures of breast cancer datasets (25) published by the authors. Detailed information on each dataset can be found in Supplementary III-Sheet 1&2. |

| **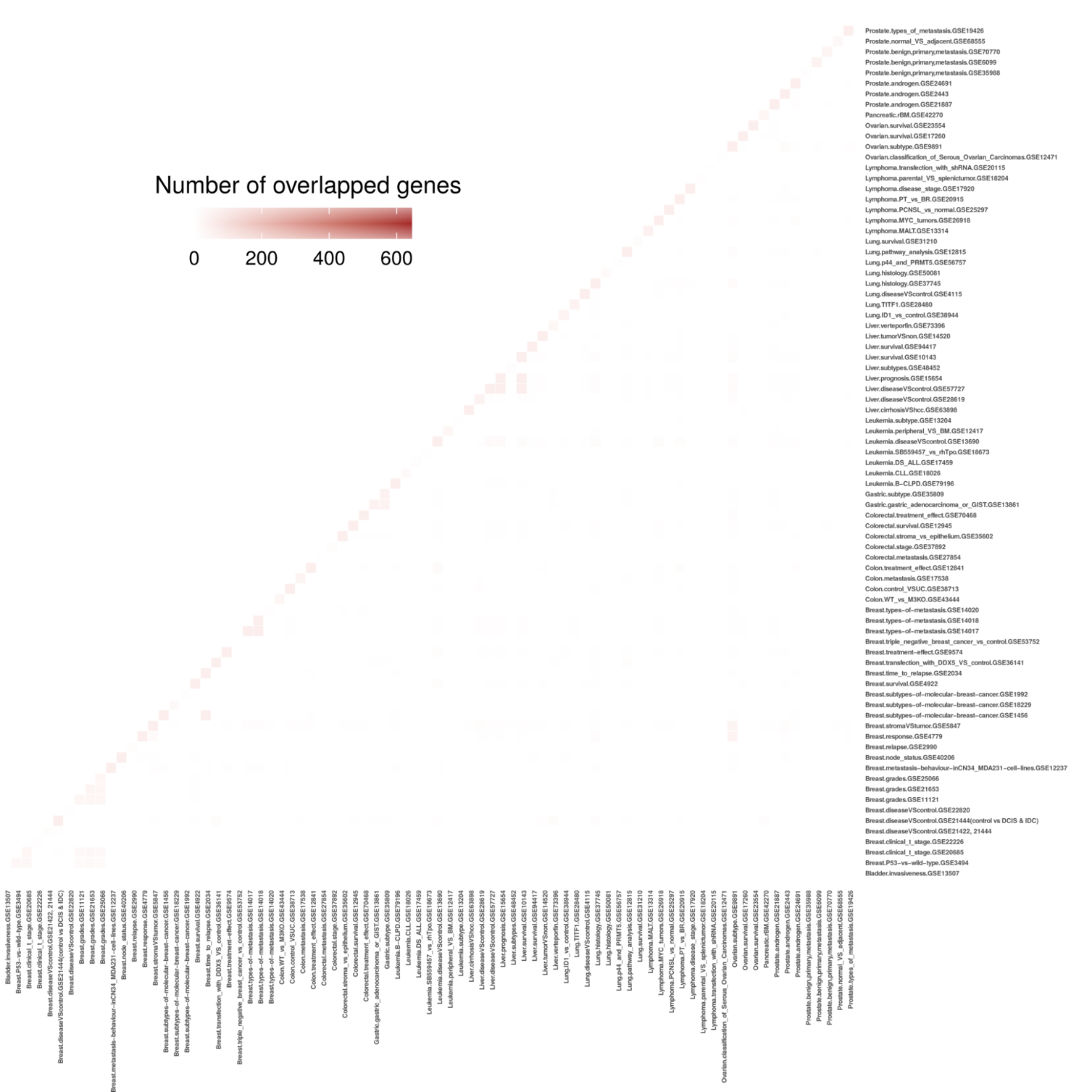** |
| --- |
| **Figure S3. Heatmap of all collected author signatures.** Heatmap showing overlapping gene signatures of all (105) cancer datasets reported by the authors. Detailed information on each dataset can be found in Supplementary III-Sheet 1&2. There is nearly no overlap between signatures. Three signatures were removed from this heatmap as they have lengths of between 1000 to 3000 genes, so including them would lead to loss of all contrast in the heatmap. |

| **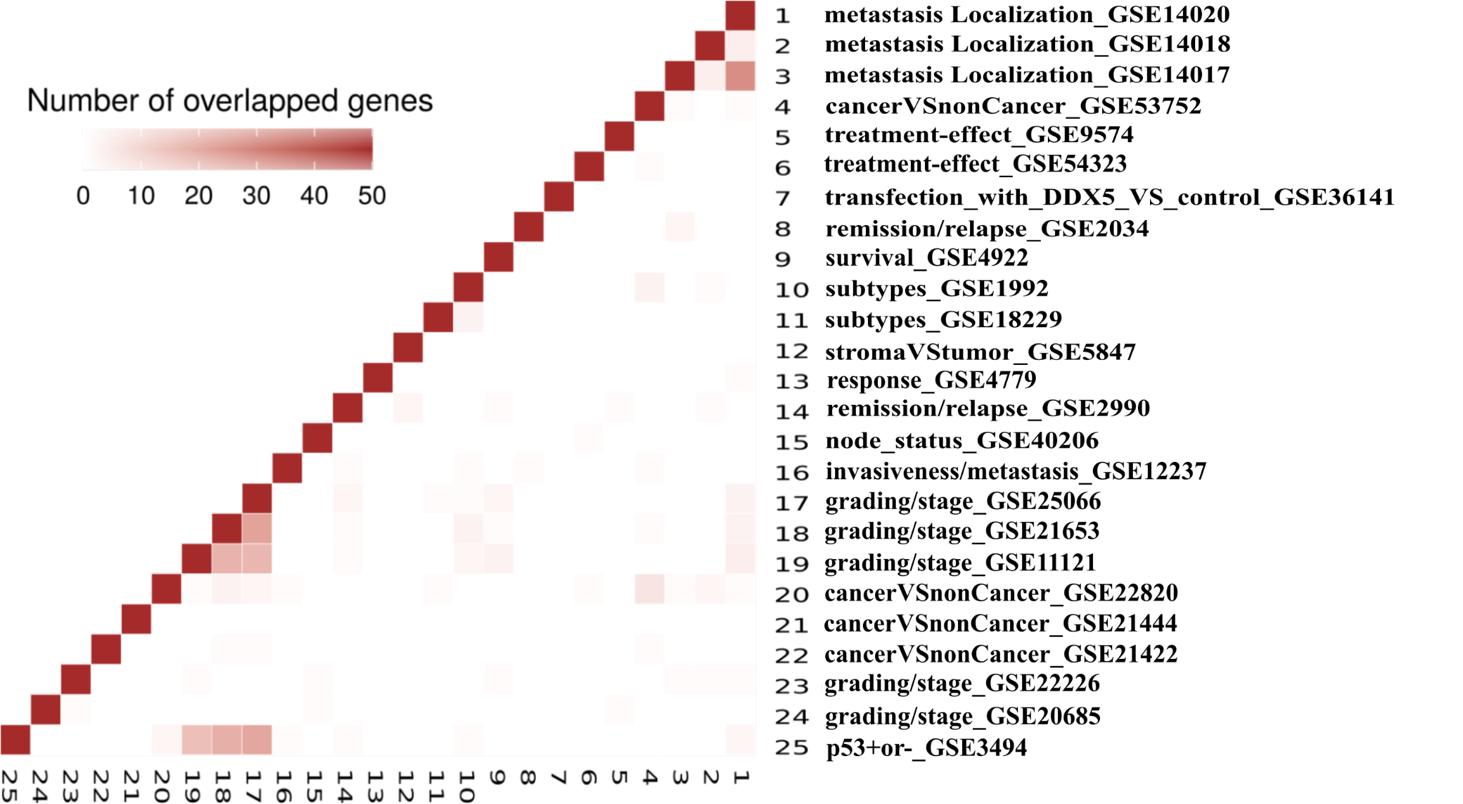** |
| --- |
| **Figure S4. Heatmap of overlapped gene signatures from breast cancer datasets using the standard correlation method and selecting the best 50 in terms of the association p-value.** Heatmap showing overlapping gene signatures of breast cancer datasets using standard correlation (see methods). The upper right (Y-axis) shows the specific phenotype and GEO accession ID of each dataset. The X-axis indicates the number of identical datasets used for the Y-axis. There is hardly any overlap among signatures even though signatures are generated with the same method. Detailed information on each dataset can be found in Supplementary III-Sheet 1&2. |

| **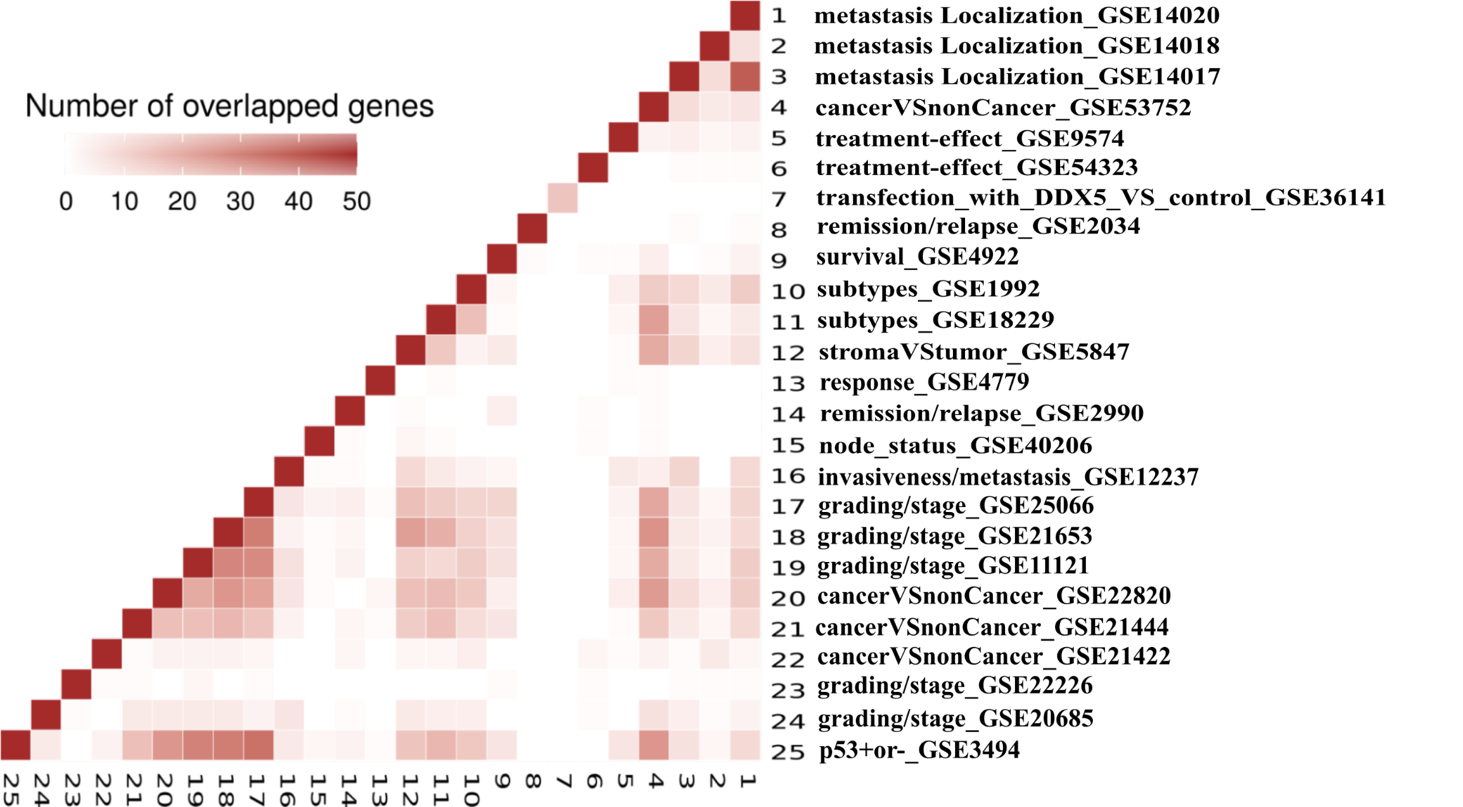** |
| --- |
| **Figure S5. The heatmap of overlapped gene signatures from breast cancer datasets by using NetRank.** Heatmap showing overlapping gene signatures of breast cancer datasets using NetRank. With NetRank, signatures do overlap even across different phenotypes. Detailed information on each dataset can be found in Supplementary III-Sheet 1&2. |

| **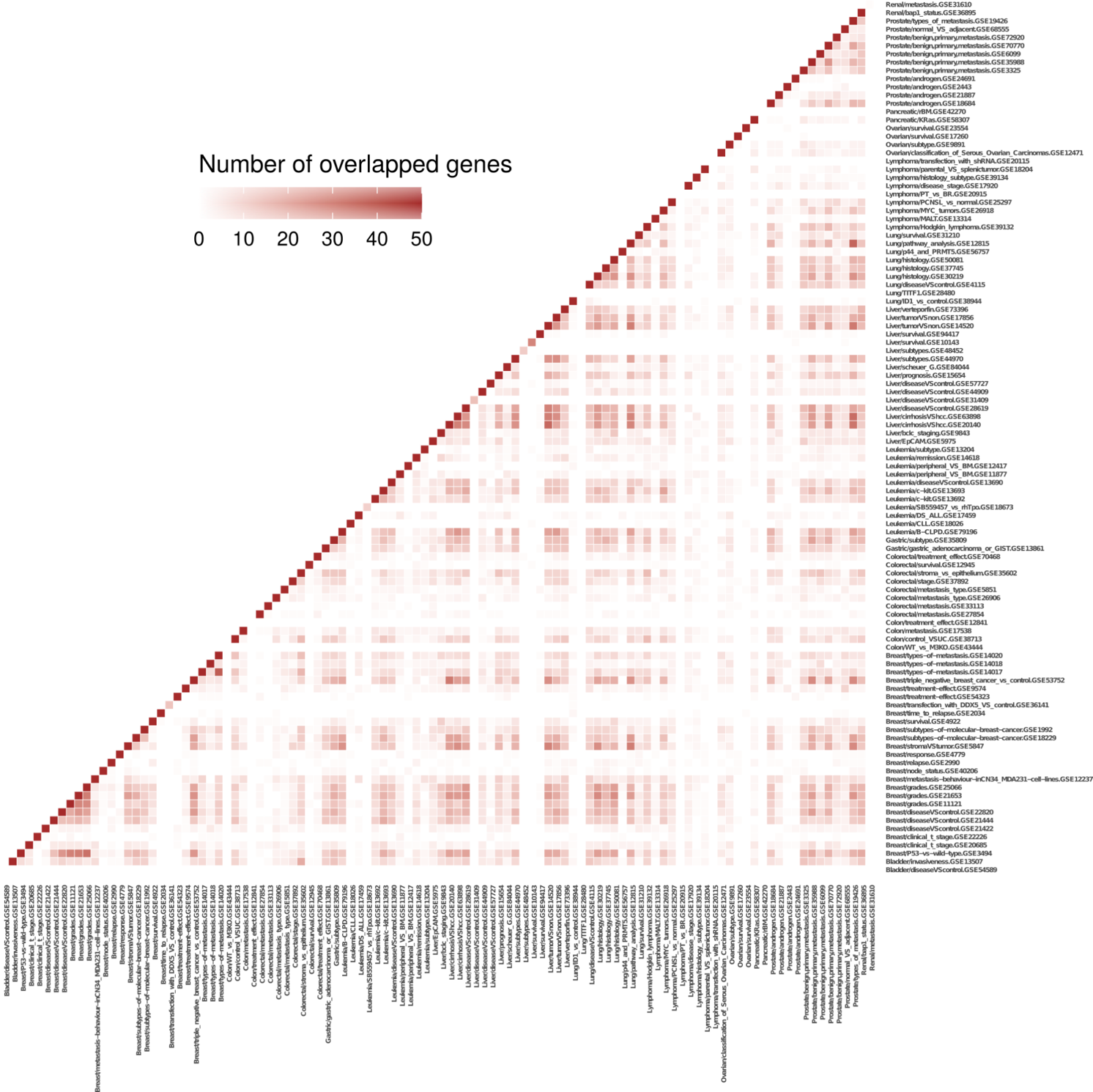** |
| --- |
| **Figure S6. Heatmap of overlapped gene signatures from all (105) datasets by using NetRank.** The X-axis and Y-axis show the signature phenotypes and GEO accession ID of each dataset. With NetRank, signatures do overlap even across different phenotypes and cancer types. Detailed information on each dataset can be found in Supplementary III-Sheet 1&2. |

**
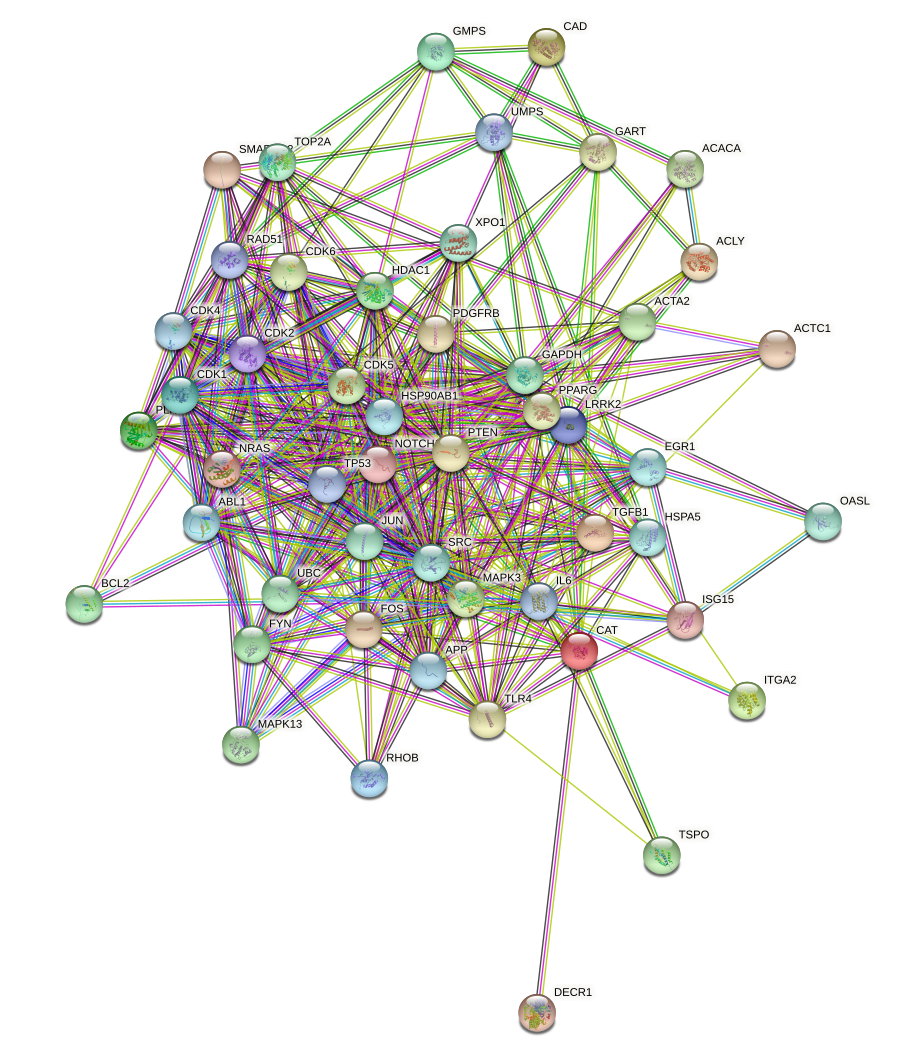
**

**Figure S7: Network analysis of universal NetRank signature in String database Version 11.5.** The query was done on September 22, 2021.

**Table S1: GEO accession ID of the analyzed datasets in this study.**

| **CANCER TYPE** | **GEO accession ID of datasets** |
| --- | --- |
| **BREAST** | GSE21422, GSE21444, GSE22820, GSE12237, GSE36141, GSE14020, GSE14017, GSE14018, GSE1992, GSE2034, GSE2990, GSE18229, GSE22226, GSE5847, GSE25066, GSE11121, GSE4922, GSE53752, GSE3494, GSE4779, GSE40206, GSE21653, GSE20685, GSE9574, GSE54323 |
| **LIVER** | GSE84044, GSE10143, GSE63898, GSE94417, GSE28619, GSE48452, GSE73396, GSE15654, GSE57727, GSE44970, GSE44909, GSE31409, GSE14520, GSE9843, GSE20140, GSE17856, GSE5975 |
| **PROSTATE** | GSE6099, GSE3325, GSE68555, GSE70770, GSE72920, GSE19426, GSE18684, GSE21887, GSE2443, GSE35988, GSE24691 |
| **LEUKEMIA** | GSE79196, GSE11877, GSE13204, GSE17459, GSE14618, GSE18026, GSE18673, GSE13693, GSE13692, GSE13690, GSE12417 |
| **LUNG** | GSE31210, GSE50081, GSE56757, GSE30219, GSE38944, GSE37745, GSE12815, GSE4115, GSE28480 |
| **LYMPHOMA** | GSE26918, GSE39134, GSE39132, GSE25297, GSE20115, GSE20915, GSE18204, GSE17920, GSE13314 |
| **COLORECTAL** | GSE70468, GSE35602, GSE5851, GSE33113, GSE27854, GSE12945, GSE26906, GSE37892 |
| **COLON** | GSE38713, GSE43444, GSE17538, GSE12841 |
| **OVARIAN** | GSE23554, GSE9891, GSE17260, GSE12471 |
| **RENAL** | GSE31610, GSE36895 |
| **PANCREATIC** | GSE42270, GSE58307 |
| **GASTRIC** | GSE13861, GSE35809 |
| **BLADDER** | GSE13507 |
|  | |

**Table S2: Degree and betweenness centrality information of the genes of universal signature in different hallmark networks.**

Cancer Hallmarks: 1: Sustaining proliferative signaling; 2: Evading growth suppressors; 3: Evading immune destruction; 4:

Enabling replicative immortality; 5: Tumor-promoting inflammation; 6: Activating invasion and metastasis; 7: Inducing angiogenesis; 8: Genome instability and mutation; 9: Resisting cell death; 10: Reprogramming energy metabolism.

| **Gene Symbol** | **CI** | 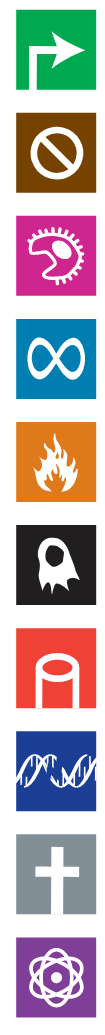  **1** | 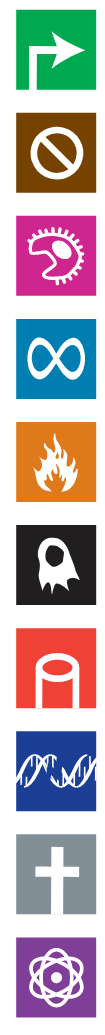  **2** | 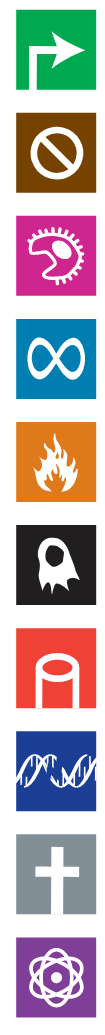  **3** | 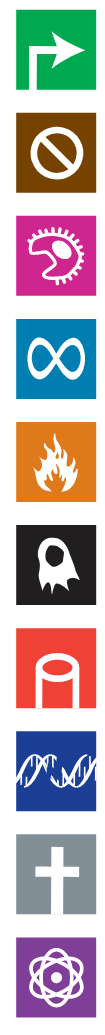  **4** | 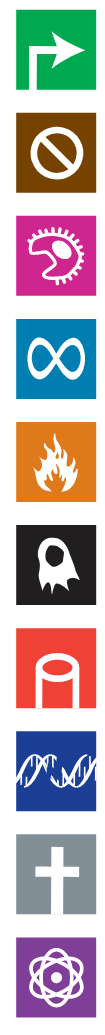  **5** | 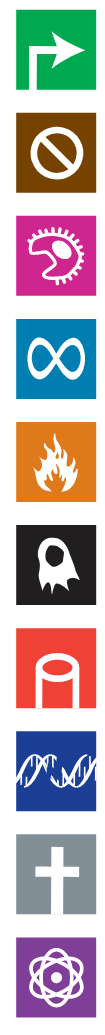  **6** | 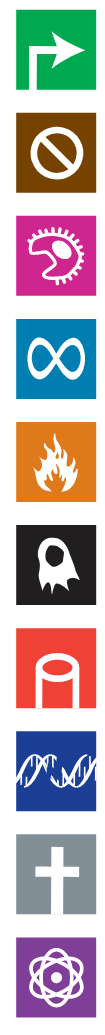  **7** | 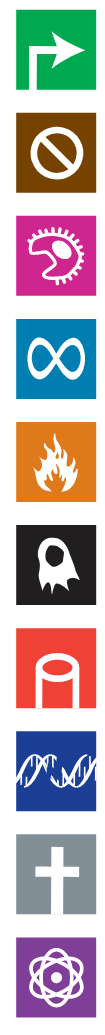  **8** | 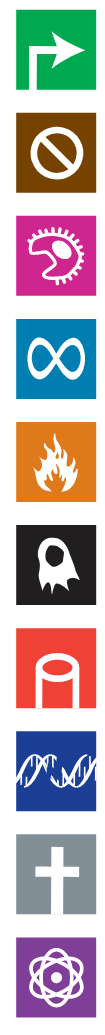  **9** | 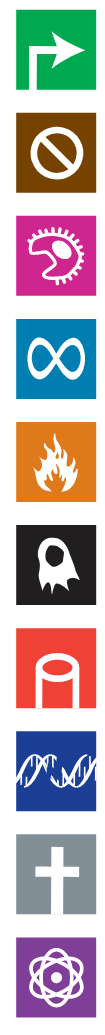  **10** |
| --- | --- | --- | --- | --- | --- | --- | --- | --- | --- | --- | --- |
| TGFB1 | Degree | 131 | 89 |  | 61 |  | 119 | 81 |  | 95 | 30 |
|  | Betweenness | 5.1*10-3 | 1.1*10-2 |  | 1.8*10-2 |  | 6.3*10-3 | 3.1*10-2 |  | 6*10-3 | 1.1*10-3 |
| IL6 | Degree | 144 |  | 60 |  | 56 |  | 99 |  | 138 | 32 |
|  | Betweenness | 4*10-3 |  | 1.5*10-2 |  | 1.1*10-2 |  | 2.1*10-2 |  | 7*10-3 | 1.8*10-3 |
| CAT | Degree |  |  |  |  |  |  |  |  | 11 | 17 |
|  | Betweenness |  |  |  |  |  |  |  |  | 2.3*10-3 | 1*10-4 |
| PDGFRB | Degree | 156 | 107 |  |  |  | 143 | 72 |  | 132 | 48 |
|  | Betweenness | 3.0*10-3 | 6.5*10-3 |  |  |  | 3.8*10-3 | 8*10-3 |  | 5.4*10-3 | 2.3*10-3 |
| PPARG | Degree | 105 |  |  |  |  |  |  |  |  | 43 |
|  | Betweenness | 1.9*10-3 |  |  |  |  |  |  |  |  | 5.6*10-3 |
| MAPK13 | Degree | 359 |  | 136 | 82 | 144 | 311 | 125 |  | 261 | 119 |
|  | Betweenness | 2.2*10-2 |  | 1.7*10-2 | 1.7*10-2 | 1.9*10-2 | 2.2*10-2 | 2*10-2 |  | 1.6*10-2 | 2.1*10-2 |
| BCL2 | Degree | 128 | 70 |  |  | 65 | 93 |  |  | 127 |  |
|  | Betweenness | 1.4*10-3 | 2.6*10-3 |  |  | 6.3*10-3 | 1.4*10-3 |  |  | 7.8*10-3 |  |
| RAD51 | Degree |  |  |  |  |  |  |  | 36 |  |  |
|  | Betweenness |  |  |  |  |  |  |  | 2.5*10-2 |  |  |
| TP53 | Degree | 252 | 149 |  | 77 |  | 200 | 88 | 82 | 242 | 107 |
|  | Betweenness | 1.5*10-2 | 3*10-2 |  | 5*10-2 |  | 1.3*10-2 | 3.6*10-2 | 318 | 43 | 44 |
| SRC | Degree | 305 |  | 162 |  | 165 | 304 | 123 |  | 227 |  |
|  | Betweenness | 1.4*10-2 |  | 4.7*10-2 |  | 4.8*10-2 | 2.3*10-2 | 2.8*10-2 |  | 1.8*10-2 |  |
| JUN | Degree | 213 |  | 102 | 61 | 99 | 152 |  |  | 173 |  |
|  | Betweenness | 8.5*10-3 |  | 1.6*10-2 | 1.4*10-2 | 1.7*10-2 | 5.2*10-3 |  |  | 1.3*10-2 |  |
| ITGA2 | Degree | 89 |  |  |  | 45 | 96 | 31 |  | 74 |  |
|  | Betweenness | 2*10-3 |  |  |  | 9.3*10-4 | 2.6*10-3 | 4.8*10-3 |  | 1*10-3 |  |
| FYN | Degree | 197 |  | 130 |  |  | 200 |  |  | 149 |  |
|  | Betweenness | 5.7*10-3 |  | 2.2*10-2 |  |  | 1.2*10-2 |  |  | 8.2*10-3 |  |
| MAPK3 | Degree | 359 | 190 | 136 | 82 | 144 | 311 | 125 |  | 261 | 119 |
|  | Betweenness | 2.2*10-2 | 4.1*10-2 | 1.7*10-2 | 1.7*10-2 | 1.9*10-2 | 2.2*10-2 | 2*10-2 |  | 1.6*10-2 | 2.2*10-2 |
| PLK1 | Degree |  |  |  |  |  |  |  |  | 129 | 55 |
|  | Betweenness |  |  |  |  |  |  |  |  | 5*10-3 | 3.2*10-3 |
| CDK6 | Degree | 109 | 68 |  |  |  |  |  | 29 | 116 |  |
|  | Betweenness | 1*10-3 | 2.2*10-3 |  |  |  |  |  | 5.5*10-3 | 3.4*10-3 |  |
| GAPDH | Degree |  |  |  |  |  |  |  |  |  | 70 |
|  | Betweenness |  |  |  |  |  |  |  |  |  | 2.3*10-2 |
| CDK1 | Degree |  |  |  |  |  | 136 |  | 51 | 189 |  |
|  | Betweenness |  |  |  |  |  | 3.3*10-3 |  | 4.2*10-2 | 1.*10-3 |  |
| FOS | Degree | 162 |  | 76 |  | 73 | 128 |  |  | 126 |  |
|  | Betweenness | 4.4*10-3 |  | 1.5*10-2 |  | 1.4*10-2 | 5.4*10-3 |  |  | 8.1*10-3 |  |
| CDK2 | Degree | 171 |  |  |  |  |  |  | 56 | 187 | 106 |
|  | Betweenness | 2.9*10-3 |  |  |  |  |  |  | 8.2*10-2 | 8.2*10-3 | 3.2*10-2 |
| NRAS | Degree | 204 | 142 | 72 | 67 | 63 | 180 | 85 |  | 133 | 67 |
|  | Betweenness | 7.3*10-3 | 2*10-2 | 2.5*10-3 | 1.2*10-2 | 2.1*10-3 | 8.3*10-3 | 1.5*10-2 |  | 2*10-3 | 3.5*10-3 |
| CDK4 | Degree | 131 |  | 39 |  |  | 96 |  | 45 | 132 |  |
|  | Betweenness | 1.5*10-3 |  | 5.2*10-4 |  |  | 1.2*10-3 |  | 2*10-2 | 5*10-3 |  |
| HSP90AB1 | Degree | 143 |  |  |  |  |  |  |  | 126 |  |
|  | Betweenness | 3*10-3 |  |  |  |  |  |  |  | 4.3*10-3 |  |
| PTEN | Degree | 133 | 74 |  |  |  | 120 |  | 14 | 111 | 73 |
|  | Betweenness | 3.2*10-3 | 5.7*10-3 |  |  |  | 4.6*10-3 |  | 8*10-3 | 5*10-3 | 1*10-3 |
| ACLY | Degree |  |  |  |  |  |  |  |  |  | 34 |
|  | Betweenness |  |  |  |  |  |  |  |  |  | 8.6*10-3 |
| ACACA | Degree |  |  |  |  |  |  |  |  |  | 53 |
|  | Betweenness |  |  |  |  |  |  |  |  |  | 1.1*10-2 |
| HDAC1 | Degree | 128 | 85 |  |  |  |  |  |  |  |  |
|  | Betweenness | 2*10-3 | 5*10-3 |  |  |  |  |  |  |  |  |
| NOTCH1 | Degree | 143 | 95 |  |  |  |  |  |  |  |  |
|  | Betweenness | 5*10-3 | 1*10-3 |  |  |  |  |  |  |  |  |
| UBC | Degree | 249 |  |  |  |  |  |  |  |  |  |
|  | Betweenness | 1.4*10-2 |  |  |  |  |  |  |  |  |  |
| ABL1 | Degree | 172 | 105 |  |  |  | 147 |  |  | 132 |  |
|  | Betweenness | 4.5*10-3 | 6.9*10-3 |  |  |  | 4*10-3 |  |  | 5*10-3 |  |
| TLR4 | Degree | 13 |  | 19 |  | 16 | 11 | 3 |  | 10 |  |
|  | Betweenness | 3.6*10-5 |  | 2.8*10-4 |  | 2.9*10-4 | 4.6*10-5 | 6.6*10-4 |  | 7*10-5 |  |
| **SUM of Degree** | | 4196 | 1174 | 932 | 430 | 870 | 2747 | 832 | 313 | 3281 | 973 |

**Supplementary Sheets S1**

**Sheet 1: All fetched PubMed publications (3,668) using Biopython code**

<https://docs.google.com/spreadsheets/d/1NQoFMaGSbq4R0R8FvR_nTRmBBfoB5vNyJzlZ_FztOyY/edit#gid=0>

**Sheet 2: List of all filtered publications (1,288) relevant to our work interest**

<https://docs.google.com/spreadsheets/d/1NQoFMaGSbq4R0R8FvR_nTRmBBfoB5vNyJzlZ_FztOyY/edit#gid=2146485251>

**Sheet 3: All the publicly available datasets (225) downloaded from the GEO database**

<https://docs.google.com/spreadsheets/d/1NQoFMaGSbq4R0R8FvR_nTRmBBfoB5vNyJzlZ_FztOyY/edit#gid=108462343>

**Sheet 4: Record of the analyzed datasets (105) after the elimination of 120 datasets due to lower quality or lack of information required for our analysis**

<https://docs.google.com/spreadsheets/d/1NQoFMaGSbq4R0R8FvR_nTRmBBfoB5vNyJzlZ_FztOyY/edit#gid=1721797689>

**Link to the code to fetch dataset:**

[**https://colab.research.google.com/drive/1CCL8c-1oo6lVua8RUfFNXk3IIZVQ5LNG#scrollTo=9AlsThHKbFhn**](https://colab.research.google.com/drive/1CCL8c-1oo6lVua8RUfFNXk3IIZVQ5LNG#scrollTo=9AlsThHKbFhn)

**Supplementary Sheets S2**

**Sheet 1: Detailed information for all datasets (105) used in this article.**

[https://docs.google.com/spreadSheets/d/17gaEQePmBG8a0H476_XzF1LIeGUgzdVdjk64YjWGIiY/edit#gid=237356170](https://docs.google.com/spreadsheets/d/17gaEQePmBG8a0H476_XzF1LIeGUgzdVdjk64YjWGIiY/edit#gid=237356170)

**Sheet 2: Description of phenotypes that are included in this study.**

[https://docs.google.com/spreadSheets/d/17gaEQePmBG8a0H476_XzF1LIeGUgzdVdjk64YjWGIiY/edit#gid=536014769](https://docs.google.com/spreadsheets/d/17gaEQePmBG8a0H476_XzF1LIeGUgzdVdjk64YjWGIiY/edit#gid=536014769)

**Sheet 3: All PCA analysis used for Figure 5** (Comparison of selection performance of NetRank and standard correlation method). We calculated the area under the ROC curve (AUC) of the best principal component in three scenarios. First, by using standard correlation only (without NetRank), the best 50 genes that NetRank selected with the highest NetRank score and p-value of association below 0.01; (3) AUC scores (yellow) of the 50 most overlapped genes between different cancer types (universal signature). The closer the AUC to 1, the better the predictive model. [https://docs.google.com/spreadSheets/d/17gaEQePmBG8a0H476_XzF1LIeGUgzdVdjk64YjWGIiY/edit#gid=237356170](https://docs.google.com/spreadsheets/d/17gaEQePmBG8a0H476_XzF1LIeGUgzdVdjk64YjWGIiY/edit#gid=237356170)

**Sheet 4: Mutation frequencies of 50 genes from The Cancer Genome Atlas - Genomic Data Commons Data Porta**l.

[https://docs.google.com/spreadSheets/d/17gaEQePmBG8a0H476_XzF1LIeGUgzdVdjk64YjWGIiY/edit#gid=936333995](https://docs.google.com/spreadsheets/d/17gaEQePmBG8a0H476_XzF1LIeGUgzdVdjk64YjWGIiY/edit#gid=936333995)

**Sheet 5: Drug names for the targetable proteins within the proposed universal signature.**

[https://docs.google.com/spreadSheets/d/17gaEQePmBG8a0H476_XzF1LIeGUgzdVdjk64YjWGIiY/edit#gid=536014769](https://docs.google.com/spreadsheets/d/17gaEQePmBG8a0H476_XzF1LIeGUgzdVdjk64YjWGIiY/edit#gid=536014769)

**Sheet 6:** **Number of datasets and cancer types that voted for universal signature.** As a result of NetRank analysis, the first 50 genes expressed in the highest number of cancer types were accepted as universal signatures. <https://docs.google.com/spreadsheets/d/17gaEQePmBG8a0H476_XzF1LIeGUgzdVdjk64YjWGIiY/edit#gid=1392446066>

**Supplementary Sheets 3**

**Sheet 1-13: Signatures comprising 50 genes found for each of the 13 cancer types using NetRank along with the number of times a particular gene appears in different datasets of a specific cancer type.**

<https://docs.google.com/spreadsheets/d/1CEqcz8PqYbCmWUR0V8pzsPYBAQoU9NZ6JuK2G8V66tY/edit#gid=0>
